# Vertical Transmission of Zika Virus by Florida *Aedes aegypti* and *Ae. albopictus*

**DOI:** 10.3390/insects14030289

**Published:** 2023-03-16

**Authors:** Rebecca A. Zimler, Barry W. Alto

**Affiliations:** Florida Medical Entomology Laboratory 200 9th St. S.E., Entomology and Nematology Department, Institute of Food and Agricultural Sciences, University of Florida, Vero Beach, FL 32962, USA

**Keywords:** vertical transmission, filial infection, invasive mosquitoes, Zika virus transmission, emerging pathogen, epidemiology

## Abstract

**Simple Summary:**

*Aedes aegypti* and *Ae. albopictus* mosquitoes are competent transmitters of Zika virus (ZIKV) and are widely distributed throughout the state of Florida. Investigations of the long-term maintenance of the virus that allows outbreaks to persist in adverse environmental conditions are limited. One mechanism for arboviral maintenance in nature is vertical transmission (VT) of a virus passed directly from parent to offspring during reproduction. This study assesses the potential of Florida *Ae. aegypti* and *Ae. albopictus* to vertically transmit ZIKV. To address this gap in our understanding of this critical risk parameter, we orally exposed Florida F3 generation *Ae. aegypti* and *Ae. albopictus* to ZIKV infected blood meals using a dose of ZIKV within the range of viremia levels experienced by infected humans. We observed low VT in both *Ae. aegypti* (1.1–3.2%) and *Ae. albopictus* (0–0.3%) mosquitoes; despite imbibing infected blood at titers that yielded high susceptibility to infection and modest horizontal transmission rates. Filial infection rates; testing individual mosquitoes; for *Ae. aegypti* and *Ae. albopictus* were 6–10% and 0–6.4%, respectively. Both invasive *Stegomyia* mosquitoes were capable of vertically transmitting ZIKV under laboratory conditions; and approximately 5% of female progeny of *Ae. aegypti* were capable of transmitting ZIKV upon first bite.

**Abstract:**

The Zika virus pandemic of 2015, with mosquitoes *Aedes aegypti* and *Ae. albopictus* as the putative vectors, prompted public health concerns and the need to improve our understanding of both the horizontal and vertical transmission of Zika virus. Local transmission is especially concerning for Florida, where these two mosquito species are abundant and widespread throughout much of the year. Here, we evaluate the relative vertical transmission and filial infection rate of progeny of Florida *Ae. aegypti* and *Ae. albopictus* following ingestion of infected blood by parental mosquitoes at either 6 or 7 log_10_ plaque forming units/mL of Zika virus. Florida *Ae. aegypti* exhibited higher rates of disseminated infection than *Ae. albopictus*, consistent with other studies indicating greater permissibility of Zika virus in *Ae. aegypti.* We observed low vertical transmission in both *Ae. aegypti* (1.1–3.2%) and *Ae. albopictus* (0–0.3%) mosquitoes, despite imbibing infected blood at titers that yielded high susceptibility to infection and modest horizontal transmission rates. Filial infection rates, testing individual mosquitoes for *Ae. aegypti* and *Ae. albopictus*, were 6–10% and 0–6.4%, respectively. Both these invasive *Stegomyia* mosquitoes were capable of vertically transmitting Zika virus under laboratory conditions, and approximately 5% of female progeny of *Ae. aegypti* were capable of transmitting Zika virus upon first bite.

## 1. Introduction

There are gaps in our understanding about Zika virus (ZIKV) and its interactions with putative mosquito vectors and the environment that contribute to its epidemiology. Unlike most other arboviruses, ZIKV appears to be more permissive in its human host, as indicated by a relatively high number of non-mosquito related routes of transmission (sexual, postnatal mother-to-child, and blood transfusion). Variation in the susceptibility to infection and horizontal transmission of arboviruses by mosquito vectors can be influenced by intrinsic (viral and mosquito genetics) and extrinsic (infectious dose, temperature) factors [1,2,3,4]. Similarly, we predict that intrinsic and extrinsic factors influence rates of vertical transmission, whereby there is maternal transfer of arbovirus infection to offspring during the gonotrophic cycle.

Vertical transmission of ZIKV from the female mosquito vector to offspring may serve as a mechanism for persistence of ZIKV between seasons when there are low rates of infection in humans and environmental conditions are adverse for horizontal transmission (e.g., drought, low numbers of susceptible hosts). Putative vectors of ZIKV in subgenus *Stegomyia* of *Aedes* include mosquitoes with desiccation resistant eggs, and some species can undergo diapause (photoperiod-induced diapausing eggs laid by the female parent in *Ae. albopictus*), life history adaptations that are predicted to facilitate transmission of the virus from mosquito parent to offspring. The potential for vertical transmission of ZIKV by populations of Florida *Ae. aegypti* and *Ae. albopictus* has been understudied, and vertical transmission may contribute to ZIKV epidemiology under some environmental conditions. Thangamani and colleagues [5] suggests that vertical transmission is possible in *Ae. aegypti*. However, due to the low sample size and pooling of samples, the vertical transmission potential is still not clear. *Aedes aegypti* from Brazil have been identified as vectors with the ability to vertically transmit ZIKV; however, vertical transmission rates in *Ae. albopictus* were not examined [6]. Furthermore, the percentage of the infected female’s offspring that become infected (filial infection rate) is completely unknown. Using a single test (pooling all offspring into a single group) to determine vertical transmission allows for the calculation of a minimum infection rate (assuming at least one of the offspring is infected). However, the minimum infection rate is a crude measurement of the relative importance of vertical transmission to the epidemiology of arboviruses. On a per capita basis, vertically infected mosquitoes strongly contribute to transmission because they may be capable of transmitting the virus upon first bite. In contrast, horizontally infected mosquitoes must first become infected through ingestion of at least one infectious blood meal and complete an extrinsic incubation period (time from acquisition of infection with a pathogen until transmission is possible) before transmission is possible. Thus, a horizontally infected adult mosquito could be infectious for a much shorter period of its lifespan than a vertically infected individual.

The objective of this study is to assess the potential of *Ae. aegypti* and *Ae. albopictus* from Florida to vertically transmit ZIKV. Results from this study could be helpful in predicting the risk of transmission in Florida by providing more information on a mode of transmission that could allow the virus to survive through winters or droughts. Additionally, results from this study aim to provide useful information to help establish parameters for risk assessment models for ZIKV transmission in Florida.

## 2. Materials and Methods

### 2.1. Mosquitoes

Early generation progeny of *Ae. aegypti* (F3) and *Ae. albopictus* (F3) collected at immature stages from artificial containers in the field in Okeechobee, Florida, were used to evaluate the vertical transmission rates of ZIKV originating from Puerto Rico. Mosquito colonies were maintained in an insectary at the Florida Medical Entomology Laboratory in Vero Beach, Florida. Mosquito eggs were hatched and larvae reared at 28 °C with a 16:8 h light:dark (L:D) photoperiod. Larvae were fed a diet of 1:1 brewer’s yeast:liver powder mixture in plastic photo trays with a density of 150 larvae/liter of water. Pupae were transferred to water-holding cups and placed in BugDorm cages for eclosion. Adults were maintained at 28 °C with a 16:8 h L:D photoperiod, supplied with a 10% sucrose solution and water, and held until adults were 7–8 days old. Mosquitoes were allowed to freely mate within the cages.

### 2.2. Virus Isolate and Propagation

A ZIKV isolate from Puerto Rico (Asian lineage), GenBank: KU501215.1, strain PRVABC59, was used in all oral challenge experiments. This strain of ZIKV was deliberately chosen because it was responsible for outbreaks in the Americas (December 2015–2018) and has been associated with transmission of ZIKV in Florida. Virus stocks of Zika were propagated and tittered in African green monkey kidney cells (Vero76) with M199 medium supplemented with 10% fetal bovine serum, 0.2% penicillin/streptomycin, and 0.2% of the antifungal Mycostatin (Media reagents obtained from Thermo Fisher Scientific, Waltham, MA, USA). Following viral inoculation in T-175 cm^2^ flasks with Vero cells, cultures were incubated for six days, after which ZIKV was combined with defibrinated bovine blood to create infectious blood meals.

### 2.3. Per os Challenge of Mosquitoes

*Per os* infection of *Ae. aegypti* and *Ae. albopictus* was performed with 7–8-day-old female mosquitoes held in cylindrical cages (10 cm ht. × 10 cm top dia. × 7 cm bottom dia.) with mesh tops, with 30 mosquitoes per cage, corresponding to species. On the day before oral feeding challenges, mosquitoes were transferred to an incubator in the biosafety level-3 facility at the Florida Medical Entomology Laboratory in Vero Beach, Florida, USA. Mosquitoes were maintained in an incubator at 28 °C with a 16:8 h L:D photoperiod and starved of sucrose and water 12 h prior to the infectious blood meal feeding. The infectious blood meal consisted of defibrinated bovine blood (Hemostat, Dixon, CA, USA) and freshly propagated ZIKV at 7 log_10_ and 6 log_10_ PFU/mL. Separate groups of mosquitoes were offered each of the two ZIKV doses. Each plaque forming unit is assumed to have been derived from a single infectious virus, and PFU/mL estimates ZIKV/mL. Infectious blood meals were administered to mosquitoes using a Hemotek membrane feeding system (Discovery Workshop, Lancashire, UK) warmed to 37 °C, as described previously [7]. Adenosine triphosphate (ATP) at 0.005 M was added to the infectious blood meal as a phagostimulant. Mosquitoes were exposed in 1 h feeding trials to viremic blood meals containing ZIKV, after which the mosquitoes were cold-anesthetized for sorting. Fully engorged mosquitoes were returned to cages, with 30 fed mosquitoes per cage, along with the oviposition substrate.

The mosquitoes were maintained at 28 °C with a 16:8 h L:D photoperiod. Thirteen days after ingestion of infected blood, the mosquitoes were provided with a second non-infectious bloodmeal to undergo a second gonotrophic cycle and lay eggs. Mosquitoes were allowed to independently feed on defibrinated bovine blood (Hemostat, Dixon, CA, USA) with ATP at 0.005 M for 1 h. Mosquitoes were cold anesthetized, and fully engorged females were placed individually into 37 mL plastic tubes (h by d: 8 by 3 cm) fitted with a removable screen lid and oviposition substrate and allowed to oviposit for an additional six days.

### 2.4. Processing Mosquitoes and Detection of Zika Virus

At 18 days post-infection (dpi), the mesh lids were replaced with lids containing a honey-soaked filter paper card fastened to the inside of the lid to determine the incidence of horizontal transmission of ZIKV. Blue food coloring was added to the honey, providing a marker (visible in the mosquito crop) indicating that a mosquito fed on the honey and deposited saliva during feeding. The mosquitoes and cards were collected 24 h later. This methodology was used as an approximation to transmit ZIKV [8]. Mosquitoes that did not feed on the blue honey and expectorate saliva (absence of blue coloring in the crop) were not tested for ZIKV transmission. Eggs from the second gonotrophic cycle were removed 19 dpi and stored for at least one week to ensure complete embryonic development. Eggs from the second gonotrophic cycle were deliberately examined because the extrinsic incubation period may exceed the length of time for the first gonotrophic cycle [9]. All parental females of both species were tested for susceptibility to infection (bodies) and disseminated infection (legs) of ZIKV. Parental mosquitoes were cold anesthetized for the dissection of legs and stored at −80 °C until analysis. Legs that tested positive for the presence of ZIKV indicated a disseminated infection, a prerequisite for horizontal and vertical transmission. Progeny of parental females exhibiting disseminated infection were tested for vertical transmission. Mosquitoes were examined for the presence of viral RNA by quantitative (q) RT-PCR using methods described previously [10]. *Aedes aegypti* and *Ae. albopictus* F1 progeny from the second gonotrophic cycle from adult parents exhibiting disseminated infection (i.e., legs tested positive for presence of ZIKV) were reared to adulthood. Three days post-eclosion, individual female progeny from each female were examined for transmission potential of ZIKV by tests of saliva expectorates for ZIKV RNA collected in capillary tubes with immersion oil using forced salivation of immobilized mosquitoes [11]. Individual male progeny samples (whole bodies) were placed in separate tubes with 1 mL media, homogenized, and a 160 μL aliquot was used to isolate viral RNA using QIAamp Viral Mini kits, following the manufacturer’s protocol (Qiagen, Valencia, CA, USA). Mosquito samples were stored at −80 °C until testing. Samples were tested for the presence of ZIKV RNA by quantitative RT-PCR using the CFX96 Real-Time PCR Detection System (Bio-Rad Laboratories, Hercules, CA, USA) and primers and probes specific to the Asian lineage of ZIKV, using established methods [8].

### 2.5. Statistical Analysis

The percentage of infected samples was calculated by dividing the number of ZIKV positive mosquito bodies, legs, or saliva by the total number of mosquitoes sampled per treatment group. Separate analyses were performed to characterize susceptibility to infection (bodies), disseminated infection (legs), and transmission potential (saliva). Significant effects were followed by pairwise comparisons of treatments, correcting for multiple comparisons using a Bonferroni adjustment (PROC MULTTEST, SAS 9.4). Vertical transmission was calculated as the minimum infection rate of ZIKV infected progeny from female parents with ZIKV disseminated infections. Filial infection was calculated as the percent of ZIKV infected progeny from each individual infected female parent. Separate analyses were performed for *Ae. aegypti* and *Ae. albopictus* progeny and for tests of disseminated infection of female parents, vertical transmission to progeny, and filial infection of progeny by using maximum likelihood categorical analyses of contingency tables (PROC CATMOD, SAS 9.4) based on the number of mosquitoes categorized for the presence or absence of ZIKV. Sex-specific filial infection rates (male versus female) were also determined.

## 3. Results

### 3.1. Parental Infection Rate

**Figure 1:**
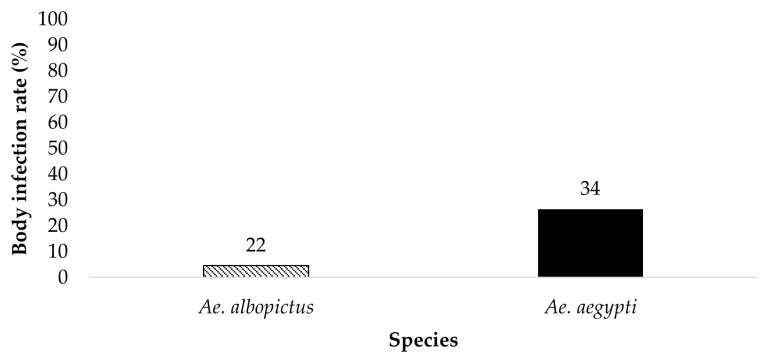
All bodies were tested for the presence of ZIKV infection. The results show the susceptibility to ZIKV infection of parental *Ae. aegypti* and *Ae. albopictus* from the treatment groups ingesting infected blood at a dose of 6 log_10_ PFU/mL. The numbers above the bars represent the number of mosquitoes tested.

**Figure 2:**
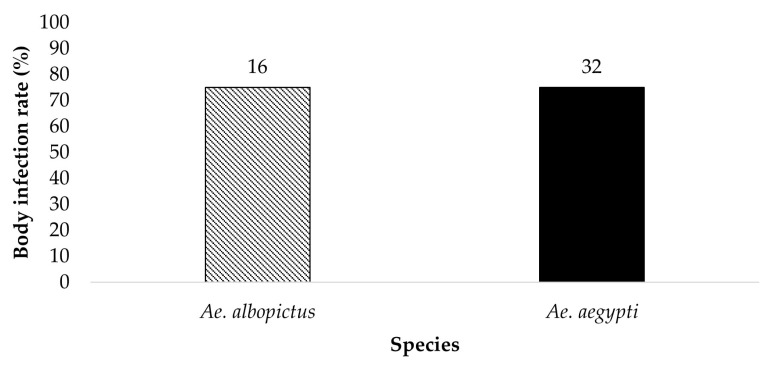
All bodies were tested for the presence of ZIKV infection. The results show the susceptibility to ZIKV infection of parental *Ae. aegypti* and *Ae. albopictus* from the treatment groups ingesting infected blood at a dose of 7 log_10_ PFU/mL. The numbers above the bars represent the number of mosquitoes tested.

All bodies from parent mosquitoes were tested for the presence of ZIKV infection to determine the susceptibility to infection 19 dpi (Figure 1 and Figure 2). *Ae. aegypti* (*n* = 34) and *Ae. albopictus* (*n* = 22) offered 6 log_10_ PFU/mL exhibited an infection rate of 26.5% and 4.5%, respectively. *Ae. aegypti* (*n* = 32) and *Ae. albopictus* (*n* = 16) offered 7 log_10_ PFU/mL showed an infection rate of 75% and 75%, respectively. The contingency table analysis did not show a significant difference between infection by species or by species according to dose interaction. A significant effect was observed between doses (Table 1). Follow-up tests indicate a higher infection rate in mosquitoes offered the 7 log_10_ PFU/mL infectious blood meal (*χ*^2^ = 29.59, *df* = 1, *p* > 0.0001).

**Figure 3:**
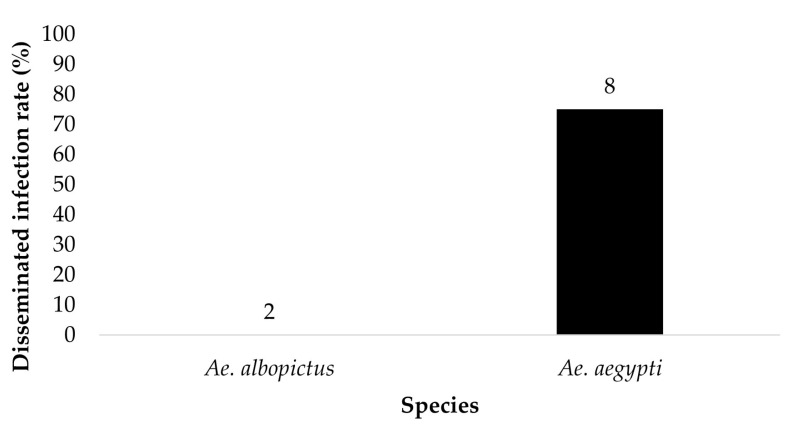
The legs of the ZIKV positive bodies (parents) were tested for the presence of ZIKV. The results show the disseminated infection of ZIKV in *Ae. aegypti* and *Ae. albopictus* from the treatment groups ingesting infected blood at a dose of 6 log_10_ PFU/mL ZIKV. Numbers above the bars represent the number of mosquitoes tested.

**Figure 4:**
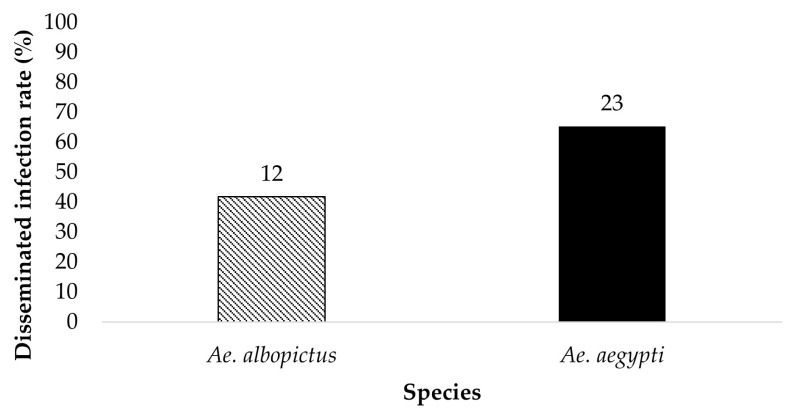
The legs of the ZIKV positive bodies (parents) were tested for the presence of ZIKV. The results show the disseminated infection of ZIKV in *Ae. aegypti* and *Ae. albopictus* for the treatment Groups ingesting infected blood at a dose of 7 log_10_ PFU/mL. Numbers above the bars represent the number of mosquitoes tested.

The legs of ZIKV positive bodies were examined for disseminated infection (Figure 3 and Figure 4). *Ae. aegypti* (*n* = 8) and *Ae. albopictus* (*n* = 2) offered 6 log_10_ PFU/mL had a dissemination rate of 75% and 0%, respectively. *Ae. aegypti* (*n* = 23) and *Ae. albopictus* (*n* = 12) offered 7 log_10_ PFU/mL had a dissemination rate of 65.2% and 41.7%, respectively. The contingency table analysis showed a marginally significant difference between infection by species (*p* = 0.06), and no significant effects of dose, or species by dose interaction (Table 1). Overall, greater numbers of *Ae. aegypti* mosquitoes exhibited disseminated infection than adult female *Ae. albopictus*.

**Figure 5:**
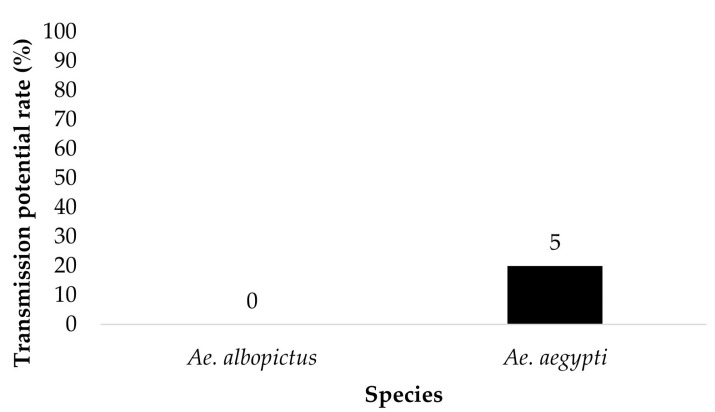
Transmission potential (saliva infection) of parental *Ae. aegypti* and *Ae. albopictus* for treatment groups ingesting infected blood at a dose of 6 log_10_ PFU/mL. Numbers above the bars represent the number of mosquitoes tested.

**Figure 6:**
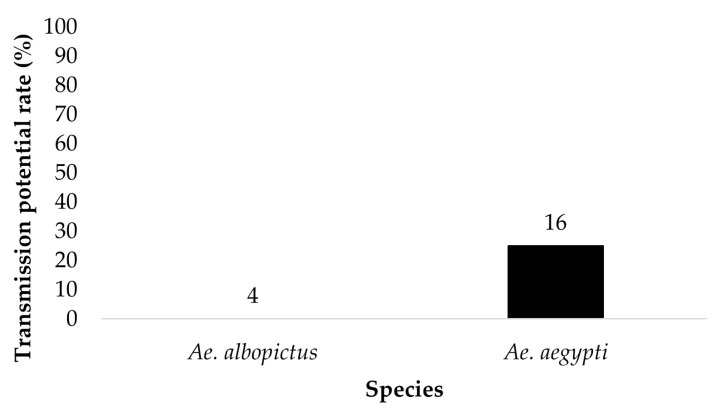
Transmission potential (saliva infection) of parental *Ae. aegypti* and *Ae. albopictus* for treatment groups ingesting infected blood at a dose of 7 log_10_ PFU/mL ZIKV infectious bloodmeal. Numbers above the bars represent the number of mosquitoes tested.

The saliva of mosquitoes that tested positive for disseminated infection and contained blue color in the crop were tested for the presence of ZIKV. *Ae. aegypti* (*n* = 5) and *Ae. albopictus* (*n* = 0) offered 6 log_10_ PFU/mL exhibited a saliva infection rate of 20% and 0%, respectively (Figure 5). *Ae. aegypti* (*n* = 16) and *Ae. albopictus* (*n* = 4) offered 7 log_10_ PFU/mL showed a saliva infection rate of 25% and 0%, respectively (Figure 6). The contingency table analysis did not show a significant difference between infection by species, dose, or species by dose interaction (Table 1).

### 3.2. Vertical Transmission Rate

The vertical transmission rate was defined as the percentage of vertically infected offspring from a population of infected females. Parent mosquitoes that ingested 7 log_10_ PFU/mL of ZIKV infected blood resulted in vertical infection rates of 3.2% (6/190) in *Ae. aegypti* and 0% (0/8) in *Ae. albopictus*. The contingency table analysis did not show a significant difference between species, sex, or a species by sex interaction (Table 2).

Parent mosquitoes offered an infectious blood meal of 6 log_10_ PFU/mL ZIKV infected blood showed vertical infection rates of 1.1% (3/275) *Ae. aegypti* and 0.3% (1/385) in *Ae. albopictus*. The contingency table analysis did not show a significant difference between species, sex, or a species by sex interaction (Table 3).

### 3.3. Filial Infection Rate

**Figure 7:**
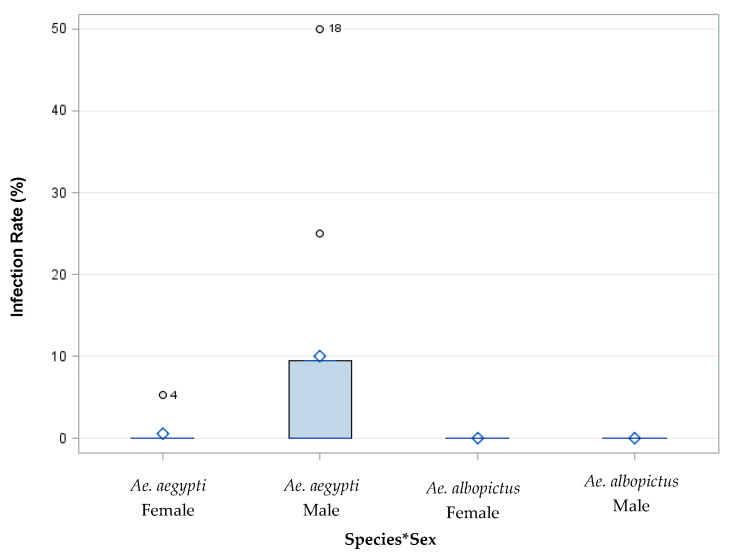
Saliva expectorates were examined for ZIKV from adult female progeny, and the whole bodies of adult male progeny were examined for the presence of ZIKV from parents fed 7 log_10_ PFU/mL ZIKV infected blood. The analysis of variance did not show a significant difference between species, sex, or their interaction. The species by sex interaction is represented by Species * Sex.

The filial infection rate was defined as the proportion of infected progeny from an individually infected female. Saliva expectorates were examined for ZIKV from adult female progeny. Additionally, the whole bodies of adult male progeny were examined for the presence of ZIKV in each treatment group. An analysis of *Ae. aegypti* females (*n* = 69), *Ae. aegypti* males (*n* = 87), *Ae. albopictus* females (*n* = 1), and *Ae. albopictus* males (*n* = 7) was performed on individual progeny from each female parent offered 7 log_10_ PFU/mL to identify the filial infection rate. The filial infection rates for *Ae. aegypti* were 6% and 0% for females and males, respectively (Figure 7). The filial infection rates for *Ae. albopictus* were 0% for both males and females. The analysis of variance did not show a significant difference between species, sex, or a species by sex interaction (Table 2, Figure 7).

**Figure 8:**
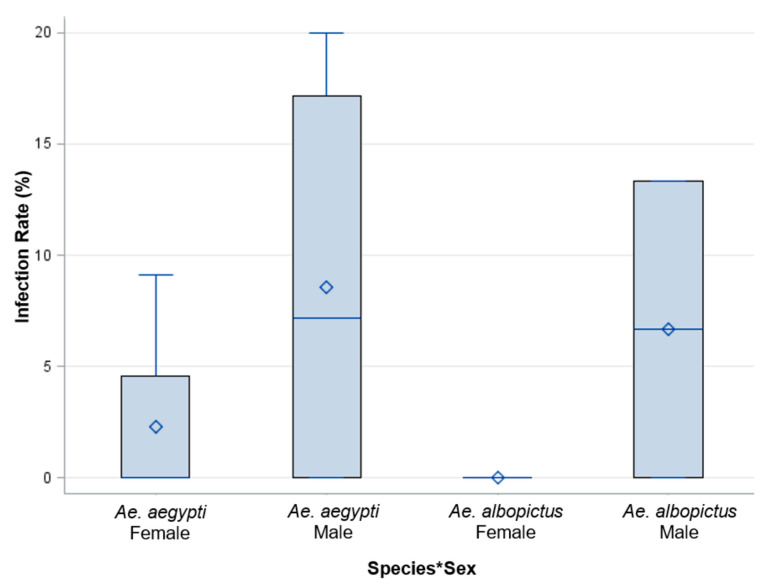
Saliva expectorates were examined for ZIKV from adult female progeny, and whole bodies of adult male progeny were examined for the presence of ZIKV from parents fed 6 log_10_ PFU/mL ZIKV infected blood. The analysis of variance did not show a significant difference between species, sex, or their interaction. The species by sex interaction is represented by Species*Sex.

An analysis of *Ae. aegypti* females (*n* = 30), *Ae. aegypti* males (*n* = 26), *Ae. albopictus* females (*n* = 5), and *Ae. albopictus* males (*n* = 15) was performed on individual progeny from each female parent offered 6 log_10_ PFU/mL to identify the filial infection rate. The filial infection rates for *Ae. aegypti* and *Ae. albopictus* were 10% and 6.4%, respectively. These results suggest a more permissive infection of ZIKV in *Ae. aegypti* than *Ae. albopictus*. However, the analysis of variance did not show a significant difference between species, sex, and a species by sex interaction (Table 3, Figure 8).

## 4. Discussion

The vertical transmission of ZIKV from female mosquito parents to offspring may serve as a mechanism for the persistence of ZIKV in an environment during adverse seasonal conditions, or when there are low numbers of susceptible hosts [5]. For example, laboratory and field evidence from Florida show that *Ae. aegypti* eggs are more desiccation tolerant than *Ae. albopictus* eggs, thus favoring the survival of *Ae. aegypti* and the associated ZIKV survival in eggs throughout a dry season, even when the adult population is reduced or does not survive [5,12]. In this study, we observed a low vertical transmission in both *Ae. aegypti* (1.1–3.2%) and *Ae. albopictus* (0–0.3%) mosquitoes, despite imbibing infected blood at titers that yielded high susceptibility to infection and modest horizontal transmission rates. The rates of vertical transmission were much lower than those of horizontal transmission for the cohorts of the fed mosquitoes, suggesting a substantial barrier for vertical transmission of ZIKV. Additionally, there was a trend showing a higher proportion of infected male progeny compared to female progeny in both treatment groups. Higher infection in males may be, in part, attributable to testing the whole-body samples, whereas saliva was tested in females. We observed a pattern of greater disseminated infection and vertical transmission of ZIKV in *Ae. aegypti* than *Ae. albopictus*, which is consistent with other studies showing greater permissiveness of ZIKV and shorter extrinsic incubation periods in *Ae. aegypti* [9,13,14].

Natural vertical transmission of ZIKV has been documented in *Ae. aegypti* larvae hatched from field collected eggs from Jojutla, Morelos, Mexico, where documented human cases of ZIKV have been reported [15]. The minimum infection rate was used to estimate vertical transmission. The infection rate in collected larvae varied between 0.28 and 0.69% from pools collected in June and November. The vertical transmission rates found in our study were higher in the *Ae. aegypti* adult progeny examined (1.1–3.2%). However, the vertical transmission rates observed in *Ae. albopictus* were lower than previously reported (F1 eggs and adults were 2.06% and 1.87%, Lai et al., 2020). Minimum infection rates are influenced by the pool sizes, and as a result, underestimate the true number of infected progeny in the population [15,16]. Another reason for the differences observed between natural vertical transmission by wild populations and that observed in laboratory studies could be explained by the higher viral concentrations used in laboratory experiments, which may allow for greater infection rates [17].

*Aedes aegypti* and *Ae. albopictus* have also been found to be able to vertically transmit ZIKV after being fed an artificial blood meal [6,18]. Further, investigation of ZIKV infection in the progeny of vertically infected female parents (filial infection rate) allows for estimates of progeny that may transmit ZIKV upon first bite. Ciota et al. [18] found filial infection rates calculated from pooled larval progeny to be 1.19% (range 0.49–2.46%) in *Ae. aegypti* and a filial infection rate of 1.18% (range 0.17–13.8%) in *Ae. albopictus.* [6] reported a minimum filial infection rate of 1 in 14.3 adult *Ae. aegypti* progeny. These filial infection rates are somewhat lower than the rates observed in our study for *Ae. aegypti* (6–10%)*;* however, the filial infection rates for *Ae. albopictus* (0–6.4%) fall within the range reported by Ciota et al. (2017). We tested individual female progeny for ZIKV infection of saliva, allowing for an improved estimate compared to the filial minimum infection rate of a pool of larval progeny. Our infection rates for the female mosquito progeny measured saliva infection, providing a more useful and improved estimate of the percent of progeny that are “infectious” upon first bite. In other words, the methodology considers barriers to ZIKV infection and transmission encountered in the progeny.

A recent study examining ZIKV and *Ae. aegypti* demonstrated the importance of considering the incubation period and number of gonotrophic cycles undergone, as both of these factors strongly influence transmission of ZIKV to progeny. Testing the ovaries of individual *Ae. aegypti* (Singapore lab colony) following ingestion of 10^5^ PFU/mL of ZIKV (strain BeH815744 from Brazil in 2015) resulted in 80% of ovary pairs infected by 3 dpi, with additional increases at 10 and 14 dpi, as well as enhanced viral load [19]. In the same study, pools of larval progeny were tested to determine minimum vertical transmission rate (assay assumes one individual is infected from a pool). Minimum vertical transmission rates at the second gonotrophic cycle were 21% at 10 dpi and 100% at 17 dpi, suggesting a high efficiency of vertical transmission of ZIKV in *Ae. aegypti* after a long incubation time. Interestingly, when mosquitoes at 17 dpi were allowed to undergo an additional gonotrophic cycle (3), the minimum vertical transmission was reduced to 76% (Manuel et al., 2020). Similarly, filial infection rates at the second gonotrophic cycle were 8.5% at 10 dpi and 66% at 17 dpi. Mosquitoes that underwent an additional gonotrophic cycle (3) exhibited a filial infection rate of 38% at 17 dpi [19]. Vertical infection of ZIKV was measured in two strains of *Ae. aegypti* from Yunnan and Hainan provinces of China [20]. The salivary glands of adult progeny of parents infected with ZIKV were tested for infection at the first and second gonotrophic cycles. The salivary gland infection rate of progeny was 16.1 to 17.1% for the first gonotrophic cycle and 2.1 to 2.3% for the second gonotrophic cycle [20]. Taken together, variation in documented filial infection rates may stem from variations in incubation time, viral titers/strain, and timing during the reproductive cycle. Conditions that promote low filial infection of ZIKV for *Ae. aegypti* suggest the need for the virus to amplify in a vertebrate host through horizontal transmission for persistence of more than a few generations [5,21]. Mathematical models of dengue transmission by *Ae. aegypti* indicate that a vertical transmission rate of 20–30% is needed for dengue virus to persist by this mode of transmission in an endemic setting. Given the same vector species *Ae. aegypti*, and a related virus, we may anticipate that similar rates would be needed for the persistence of ZIKV in instances in which horizontal transmission is low (e.g., availability of susceptible hosts, abiotic restrictions). However, a caveat should be recognized. Stabilized infections (germinal cell infection) can allow for high levels of vertically infected vectors, greatly contributing to the maintenance of pathogens [22]. For example, Tesh et al. [22] found high rates of stabilized infections in *Ae. albopictus* infected with San Angelo orthobunya virus. Models suggest that stabilized infections of only a small population could contribute to the perpetual transmission of viruses in nature by vertical transmission [22]. However, decreasing ZIKV infection rates of progeny with successive gonotrophic cycles suggest that infection wanes over the lifespan of the mosquito.

## 5. Conclusions

The results from our study indicate *Ae. aegypti* and *Ae. albopictus*, the primary and secondary vectors of ZIKV in Florida, are capable of vertically transmitting ZIKV under laboratory conditions. Additionally, a portion of female progeny of *Ae. aegypti* with vertically transmitted ZIKV are capable of transmitting ZIKV upon first bite, which may contribute to the maintenance and spread of ZIKV in nature.

## Figures and Tables

**Table 1:** Contingency table analysis of parental infection of female *Ae. aegypti* with Zika virus following an incubation period after ingestion of either 6 or 7 log_10_ PFU/mL of Zika virus infected blood. The species by dose interaction is represented by Species * Dose.

Parental Susceptibility to Infection	Factor	*df*	*χ* ^2^	*p*
	Species	1	2.06	0.12
	Dose	1	23.09	<0.0001
	Species * Dose	1	2.41	0.12
				
**Parental Disseminated Infection**	**Factor**	***df***	***χ*** **^2^**	***p***
	Species	1	3.47	0.06
	Dose	1	0.13	0.72
	Species * Dose	1	0.52	0.47
				
**Parental Saliva Infection**	**Factor**	***df***	***χ*** **^2^**	***p***
	Species	1	2.51	0.98
	Dose	1	0.83	0.36
	Species * Dose	1	0.43	0.51

**Table 2:** Contingency table analysis of saliva expectorants of adult female progeny and whole-body assays of adult male progeny from infected parental *Ae. aegypti* and *Ae. albopictus* mosquitoes offered a Zika virus infected blood meal of 7 log_10_ PFU/mL. The species by sex interaction is represented by Species * Sex.

Vertical Transmission Rate	Factor	*df*	*χ* ^2^	*p*
	Species	1	0.02	0.88
	Sex	1	0.05	0.81
	Species * Sex	1	0.03	0.85
				
**Filial Transmission Rate**	Species	1	49.91	0.57
	Sex	1	39.44	0.61
	Species * Sex	1	39.44	0.61

**Table 3:** Contingency table analysis of saliva expectorants of adult female progeny and whole-body assays of adult male progeny from infected parental *Ae. aegypti* and *Ae. albopictus* mosquitoes offered a Zika virus infected blood meal of 6 log_10_ PFU/mL. The species by sex interaction is represented by Species * Sex.

Vertical Transmission Rate	Factor	*df*	*χ* ^2^	*p*
	Species	1	0.0	0.97
	Sex	1	0.0	0.99
	Species * Sex	1	0.0	0.97
				
**Filial Transmission Rate**	Species	1	11.63	0.66
	Sex	1	112.07	0.20
	Species * Sex	1	0.09	0.96

## Data Availability

All data available is presented in the paper.
